# Sustainable and technically smart spectrophotometric determination of PAXLOVID: a comprehensive ecological and analytical performance rating

**DOI:** 10.1186/s13065-024-01275-3

**Published:** 2024-09-20

**Authors:** Sara I. Aboras, Hadir M. Maher, Nourah Z. Alzoman, Haydi S. Elbordiny

**Affiliations:** 1 Pharmaceutical Analytical Chemistry Department, Faculty of Pharmacy, Al-Mesallah, Alexandria University, Alexandria, 21521 Egypt; 2 College of Pharmacy, Department of Pharmaceutical Chemistry, King Saud University, P.O. Box 22452, 11495 Riyadh, Saudi Arabia; 3 Pharmaceutical Chemistry Department, Faculty of Pharmacy, Damanhour University, Damanhour, Egypt

**Keywords:** COVID-19, Ritonavir, Nirmatrelvir, Spectrophotometric methods, Eco-friendly, Sustainable

## Abstract

The Food and Drug Administration (FDA) authorized the administration of ritonavir (RIT)-boosted nirmatrelvir (NMV) on May 25, 2023, for the treatment of mild to moderate COVID-19 in patients who are at high risk of developing severe COVID-19. In accordance with sustainability and environmental friendliness, simple, eco-friendly, and sustainable spectrophotometric methods were established for concurrently estimating RIT and NMV in newly launched copackaged pills. The suggested solutions for resolving the spectral overlap between RIT and NMV involve the following mathematical methods: the first derivative method (^1^D), second derivative method (^2^D), and dual-wavelength zero-order method (DWZ). When ethanol was used as a green dilution solvent, the linearity range was adjusted (10–250 µg/mL) for both drugs. The procedures resulted in a high correlation coefficient (not less than 0.9996) and satisfactory levels of detection and quantification. Additionally, method validation was performed in accordance with International Council for Harmonization norms. Moreover, a detailed ecological and sustainability evaluation protocol was established to confirm the greenness and whiteness of the methods. Finally, the proposed method, along with previously reported methods for analysing NMV and RIT, were reviewed analytically and ecologically.

## Introduction

Worldwide, reports stipulate that coronavirus disease 2019 (COVID-19), caused by the severe acute respiratory syndrome coronavirus 2 (SARS-CoV-2), is still a serious illness. In a variety of global stages, particularly in the medical, cultural, and economic domains, it represents a significant burden. The SARS-CoV-2 virus is regarded as a quickly evolving pathogen that can induce a wide variety of clinical conditions, the most prevalent of which are digestive and respiratory illnesses [1]. Cough, fever, and runny nose are common symptoms in patients with respiratory dysfunctions, and these symptoms can progress to difficulty breathing and acute respiratory distress syndrome (ARDS) [2]. On the other hand, the primary problems linked to viral infection of the alimentary canal are nausea, vomiting, and diarrhoea [3]. Additionally, viral infection may be linked to thyroiditis and potential autoimmune thyroid dysfunctions [4]. Additionally, many post-COVID-19 individuals experience neuronal dysfunction and mental problems [5]. While the majority of COVID-19-infected patients recover without experiencing any noticeable consequences, certain cases are seriously impacted and necessitate hospitalization in an effort to control virus sequelae, such as ARDS, abnormal blood clots, and internal organ dysfunctions, which carry a risk of total organ failure and death [6].

Vaccines have been offered to the global market to combat such destructive viruses and reduce the likelihood and severity of viral infection. However, producing vaccines is a difficult task that is hampered by variability, affordability challenges, and incompatibility problems, in addition to economic concerns. Additionally, the effectiveness of vaccines is partially questioned because of the appearance of mutant variables [7]. Therefore, it is of public importance to research the use of therapeutic medicines to combat such viruses. Numerous treatment plans have been designed to focus on one or more viral life cycle sites and/or manage organ dysfunction caused by the virus.

**Fig. 1:**
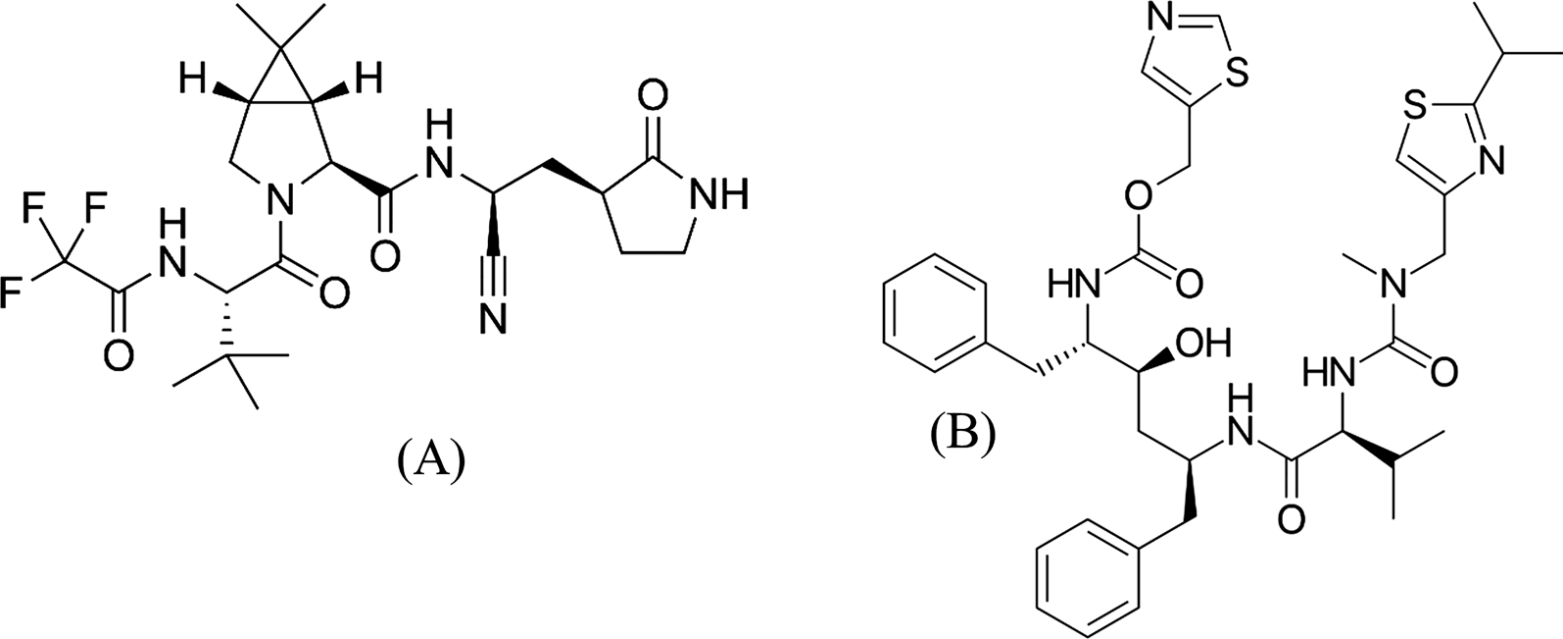
Chemical structures of (**A**) nirmatrelvir and (**B**) ritonavir

Paxlovid (nirmatrelvir copackaged with ritonavir), an effective and secure oral antiviral drug created by Pfizer, was initially announced at the beginning of 2022 and became one of the highlights of the year. The antiviral drug nirmatrelvir (NMV) (Fig. 1A) targets the 3-chymotrypsin-like cysteine protease enzyme that is present in SARS-CoV-2. NMV effectively suppresses enzymatic activity, which in turn stops virus propagation, in a range of coronaviruses. Coadministration of 300 mg NMV plus a low dose (100 mg) of the pharmacokinetic enhancer ritonavir (RIT, Fig. 1B) twice daily enhances NMV pharmacokinetics because RIT is a CYP3A4 inhibitor [8, 9]. With an 89% reduction in the likelihood of hospitalization or death within five days after the onset of symptoms, NMV/RIT has the most positive and effective therapeutic response. Owing to its high oral availability, it may also be utilized by outpatients in addition to hospitalized patients. NMV and RIT are thought to have changed the course of the COVID-19 pandemic [8–10].

After rigorous review of scientific resources, only a few methods have been described for the precise determination of Paxlovid components. NMV was analysed alone in blood [11] or in the presence of NMV degradation products [12], RIT in plasma [13], tablets [14–16] or other degradation products [16]. Various techniques have been used, including liquid chromatography‒mass spectrometry (LC‒MS) [13], high-performance liquid chromatography‒diode array detection (HPLC‒DAD) [15, 16], capillary electrophoresis (CE) [16], and high-performance thin-layer chromatography (HPTLC) [14]. Few studies have investigated the extraction efficiency of NMV and RIT via microextraction [17, 18]. However, Paxlovid^®^ has not yet been analysed spectrophotometrically. Compared with other analytical methods, spectrophotometry provides several benefits, including instrument accessibility, simplicity, cost-effectiveness, time efficiency, and ease of use, with less practical effort. Moreover, most spectrophotometric methods are of ultimate greenness, especially if a green solvent is chosen when solutions are prepared.

Spectrophotometric assays of multianalyte mixtures without previous separation often necessitate spectral mathematical treatment. Differentiating the sample's absorbance with respect to wavelength into its first, second, or higher derivative spectra can help in the separation of overlapping signals, removal of background interference, and improvement of mixture resolution, as it improves the detectability of minor spectral features. In addition, derivatization of ultraviolet (UV) spectra can improve sensitivity and specificity [19]. Moreover, the simple dual-wavelength method, whenever applicable, is an easy and useful tool in the determination of binary mixtures without requiring complex mathematical data processing. This approach is based on choosing two spectral data points whose difference depends solely on one component and does not depend on the other [20].

As in our previous publications [12, 16, 21], our goal was to reinforce the green character of pharmaceutical analysis and find alternatives to chromatographic methods, and simple, green, and reproducible spectral methods were developed for the quantitation of NMV and RIT in bulk and pharmaceutical dosage forms. The methods included first- and second-wavelength derivatives in addition to dual-wavelength derivatives for the determination of both drugs. Additionally, an analytical and ecological review was performed to compare the results achieved by the suggested methods and the reported methods in the determination of NMV and RIT. Furthermore, the principles of green analytical chemistry (GAC) and white analytical chemistry (WAC) influence the selection of environmentally friendly solvents, waste reduction, and general sustainability of analytical procedures. The integrated tools of assessment of white and green characteristics ensure that the approach created is not only technically sound but also ecologically sensitive, supporting greener and more sustainable analytical practices.

## Experimental methods

### Apparatus

A T80 UV‒Vis 1800 UV spectrophotometer from PG Instruments, UK, was used in conjunction with a computer running UVWin version 5.2.0.

### Materials and reagents

Nirmatrelvir (NMV) with a purity of 94% was graciously provided by KU Leuven Research & Development, Belgium. Ritonavir (RIT) (98% purity) was purchased from Shanghai, China's Haoyuan ChemExpress Co., Ltd.

HPLC-grade ethanol (EtOH) was purchased from Baker's, Ireland. Ultrapure PTFE syringe filters (China) measuring 0.2 µm were also used.

### Preparation of stock and standard solutions

Stock solutions of NMV or RIT, at a 1 mg/mL concentration, were prepared through the dissolution of 25 mg of each drug in a separate 25 mL volumetric flask using EtOH. These stock solutions were stored in the freezer for one week. Stock solutions were then diluted with EtOH to obtain an array of working standard solutions covering the concentration range of 10–250 µg/mL for both drugs.

### Construction of calibration curves

#### First derivative method (.^1^D)

NMV and RIT absorption spectra were measured, and first derivative (^1^D) spectra were recorded at a ∆λ of 12 nm. To create the NMV calibration graph, the signal at 222 nm (peak to zero) was measured and plotted against the NMV concentrations, as it corresponded to the zero signal of RIT. For RIT, the signal at 253 nm (peak to zero) was measured and plotted against RIT concentrations to generate the calibration graph. This working wavelength was selected since it corresponded to a zero signal of NMV. The regression equations were derived.

#### Second derivative method (^2^D)

Zero absorption spectra of NMV and RIT were generated. The second derivative spectra were computed using a ∆λ of 12 nm. For NMV determination, the signal at 222 nm (peak to zero) corresponding to the RIT zero-crossing point was measured and plotted against the NMV concentration. The signal of RIT at 253 nm (peak to zero), representing the NMV zero-crossing point, was measured and plotted against RIT concentrations to construct the corresponding calibration graph. The regression equations were also derived.

#### Dual-wavelength zero-order method (DWZ)

The wavelength range of 220–260 nm was used to scan the spectra of the working solutions. NMV was estimated by graphing the difference between the absorbance values taken at 231 and 248 nm against the corresponding concentration (the difference is 0 for RIT). Similarly, the RIT concentration was estimated by plotting the difference between the absorbance values at 251 and 272 nm against the corresponding concentration (the difference was 0 for NMV).

### Preparation and analysis of laboratory-prepared mixtures

A series of 10 mL volumetric flasks were filled with precisely measured quantities of NMV and RIT stock solutions. EtOH was used for diluting and creating mixtures with final concentrations of 10–250 µg/mL for both NMV and RIT. Three mixtures containing varying quantities of the medications under analysis were made as follows: mixtures (1), (2) and (3), which had NMV and RIT concentrations of 225 and 75 µg/mL, 100 and 100 µg/mL, and 15 and 45 µg/mL, respectively.

The previously mentioned methods were employed to determine the NMV and RIT in their laboratory-made mixtures via the procedure described in Sect. "Construction of calibration curves". A correlation regression equation was used to determine the concentration of each drug.

### Assays of dosage forms

Two 150 mg NMV tablets copacked with a 100 mg RIT tablet constitute a single dose of PAXLOVID^®^. As a result, two RIT tablets and one NMV tablet were weighed, added to the same mortar, and finely crushed. Powdered dose forms weighing 300 mg of NMV or 100 mg of RIT were combined with 50 mL of EtOH, and the mixture was then sonicated for 10 min. The mixture was subsequently filtered into a 100 mL volumetric flask. The filtrate was then brought to volume with EtOH after the remaining mixture was washed twice with 10 mL of EtOH. The final NMV and RIT concentrations of the stock solutions were 3 and 1 mg/mL, respectively. Further dilution was performed with EtOH to obtain final concentrations of NMV and RIT within the specified linearity ranges. The derived regression equations were then used to compute the corresponding concentrations.

### Greenness and whiteness assessment tools

Notably, the key to achieving analytical methods with flawless greenness is selecting the environmental solvent; therefore, the evaluation of the greenness of the solvent comprises safety, health, and environmental (SHE) impacts, all of which must be considered [22–25]. This was accomplished via the greenness index and spider diagram tools. Moreover, the green-solvent selection tool (GSST) was used to assess solvent sustainability [26].

The concept of the 3Rs (reduce, replace, and recycle) appeared to go hand in hand with the fundamentals of green analytical chemistry (GAC) [27]. They offer simplified ideas for greening analytical chemistry by reducing the sample size, waste, time, energy, and cost in addition to addressing health and environmental hazards and replacing harmful solvents with greener, biobased solvents such as water and EtOH. In this context, five methods were utilized to assess the greenness and sustainability of the whole method: the analytical eco-scale, the green analytical procedure index (GAPI), the novel analytical greenness metric (AGREE), the hexagon tool, and RGB12. The hexagon tool and RGB12 were introduced as the white analytical chemistry (WAC) subsidiary of GAC. The WAC approach involves the integration and synergy of analytical, ecological, and practical aspects [28].

## Results and discussion

### Spectrophotometric characteristics

**Fig. 2:**
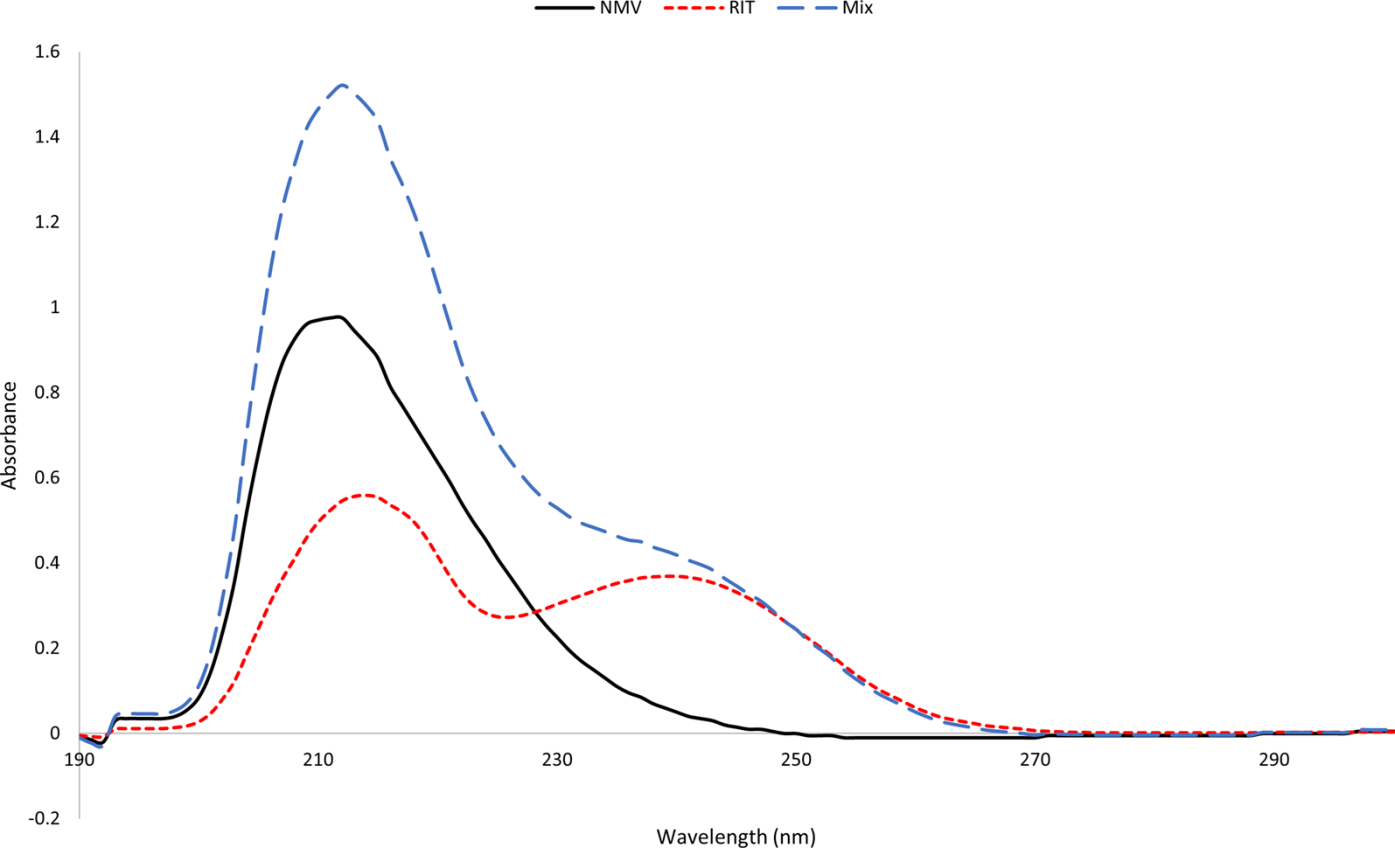
Overlay of the zero-order spectra of NMV (225 μg/mL) and RIT (75 μg/mL) and their mixture using ethanol as a blank

PAXLOVID^®^ is a combination of two antiviral medications that are taken together; hence, developing easy, rapid, and efficient methods for their simultaneous determination is urgently needed for quality control purposes. The zero-order spectra of NMV and RIT significantly overlapped, especially those of NMV, at a ratio comparable to that of the PAXLOVID^®^ dosage form, as shown in Fig. 2. As a result, simultaneous estimation of this binary combination was tricky. In this respect, three analytical methods, ^1^D, ^2^D and DWZ, are easily employed because they rely on the simple mathematics of first derivative, second derivative, and dual wavelength measurements, respectively.

Notably, we tried different dilution solvents: MeOH, EtOH, and water. The best solvent for both analytical and greenness purposes was EtOH. While water resulted in lower absorbance intensities for both NMV and RIT, MeOH resulted in better intensities but with no zero crossing points for RIT to determine NMV. On the other hand, EtOH resulted in both higher intensities and the ability to determine NMV and RIT via the proposed methods.

### Methods optimization

#### First derivative method (^1^D)

The absorbance data were differentiated with respect to λ via Excel software, which produced ^1^D (dA/dλ) signals with different maxima or minima that were extremely selective for NMV and RIT with their combination and dependent on a single analyte by zero crossing points.

**Fig. 3.**
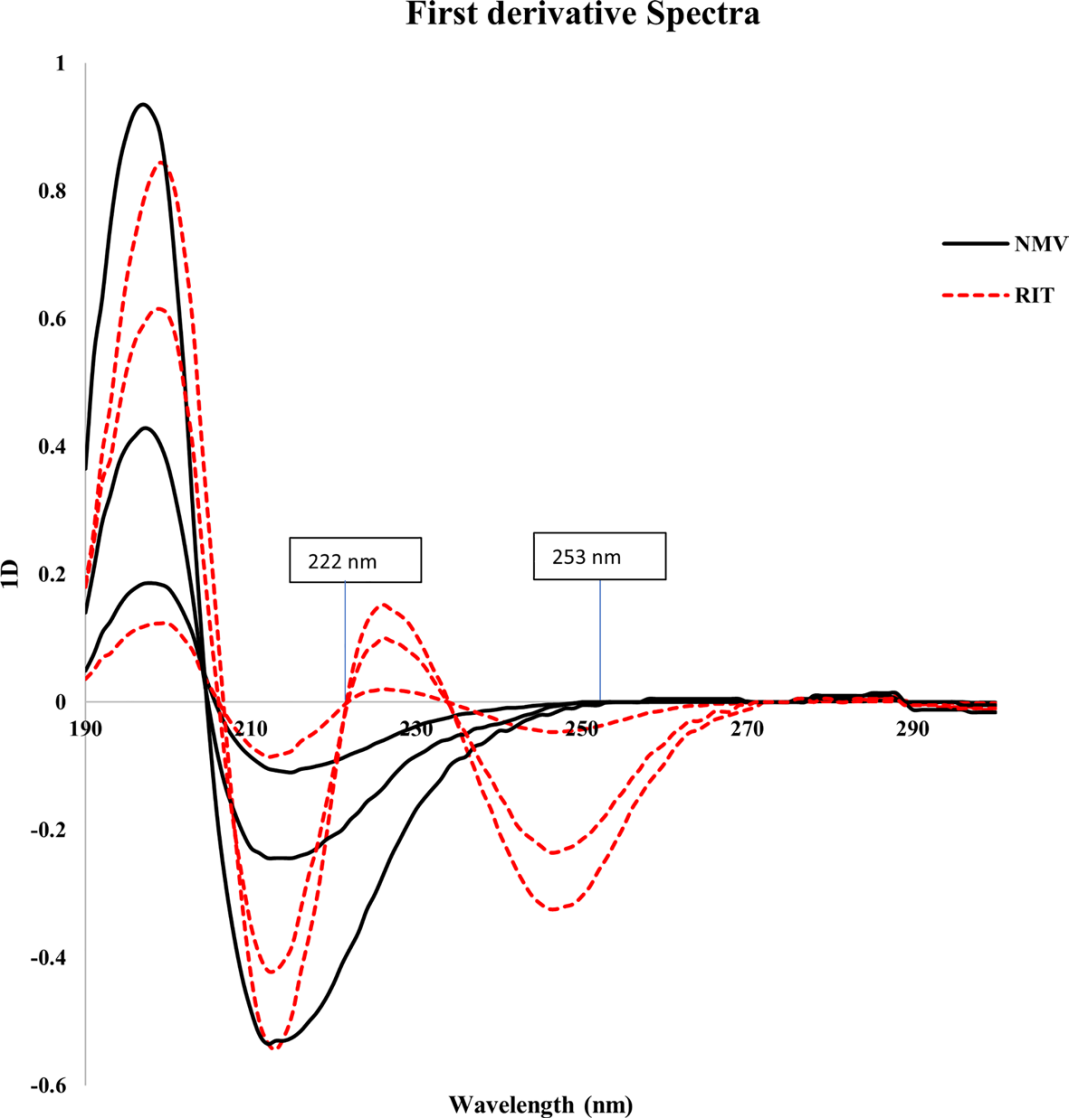
^1^D spectra of different concentrations of NMV (45, 100 and 225 μg/mL) and RIT (15, 75 and 100 μg/mL) showing zero crossing points with NMV and RIT at 253 and 222 nm, respectively

To obtain the most sensitive first derivative signals with the least amount of noise, the influence of the wavelength interval, ∆λ, was examined. A wavelength interval of 12 nm was found to be appropriate. Differentiation of the zero-order spectra resulted in ^1^D spectra with a peak at 222 nm for NMV with zero crossing with RIT (Fig. 3) and a peak at 253 nm for RIT with zero crossing with NMV (Fig. 3).

#### Second derivative method (^2^D)

**Fig. 4.**
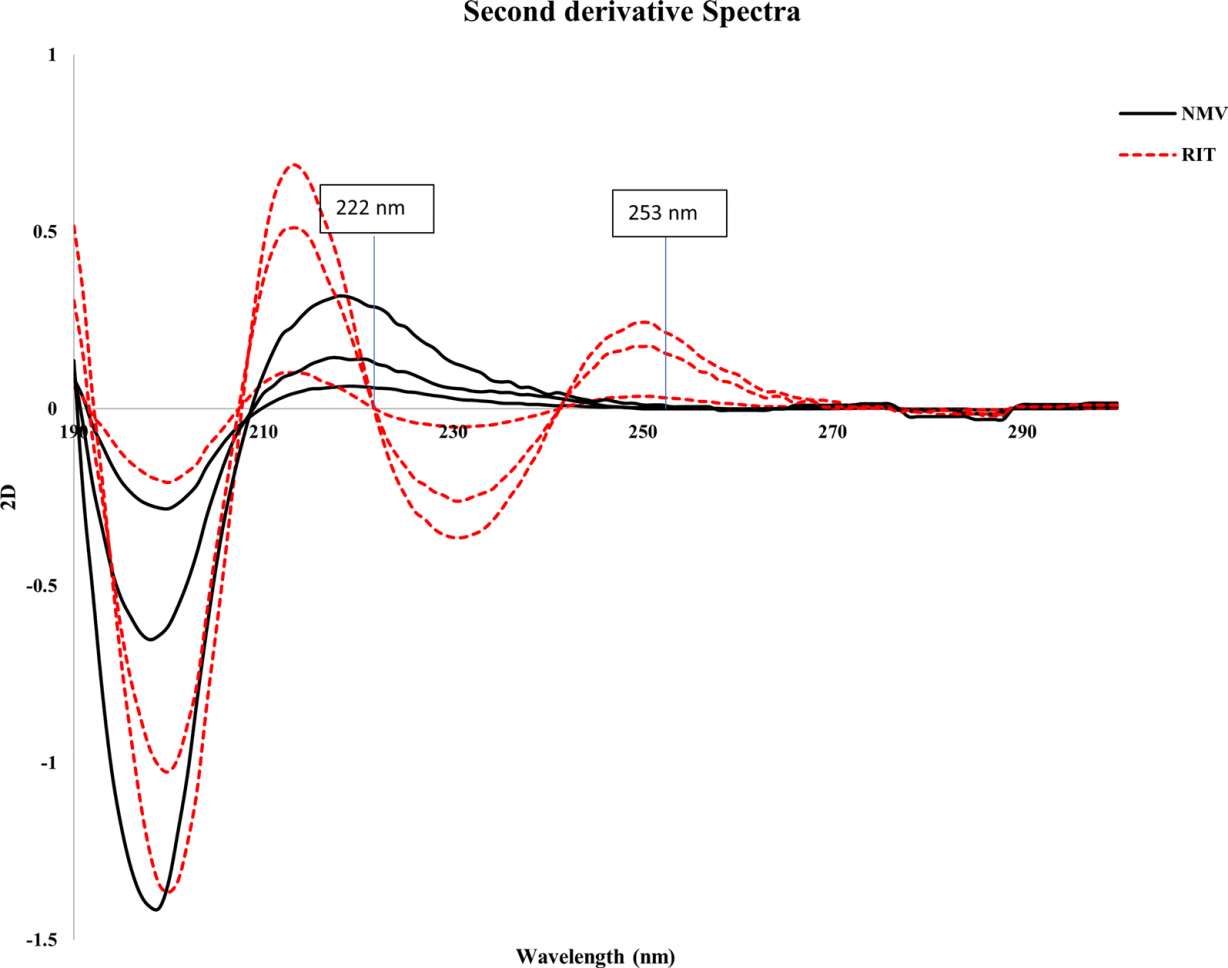
^**2**^D spectra of different concentrations of NMV (45, 100 and 225 μg/mL) and RIT (15, 75 and 100 μg/mL) showing zero crossing points with NMV and RIT at 253 and 222 nm, respectively

Another way to calculate the concentrations of NMV and RIT was by recording the second derivative spectra at a ∆λ of 12 nm against EtOH, and the d2A/dλ2 values were determined at 222 and 253 nm, respectively, and plotted against the analyte concentrations in μg/mL (Fig. 4). In addition, regression equations were created.

#### Dual-wavelength zero-order method (DWZ)

**Fig. 5:**
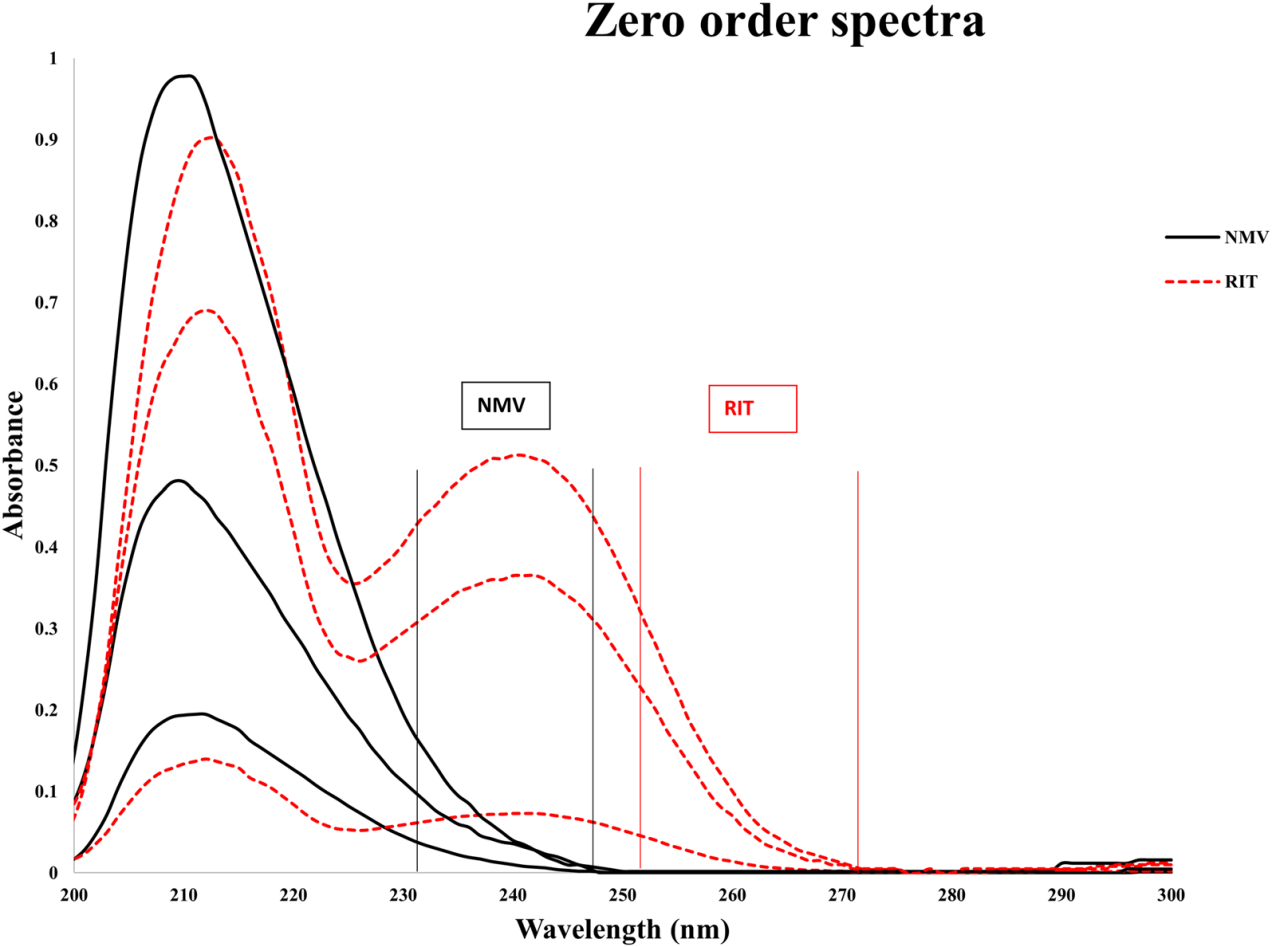
Zero-order spectra of NMV (45, 100 and 225 μg/mL) and RIT (15, 75 and 100 μg/mL) showing the selected wavelengths for DWZ when ethanol was used as a blank

As shown in Fig. 5, the use of DWZ requires the selection of two wavelengths from the absorption spectra that exhibit notable differences for one component and zero differences for the other. In this case, there is no interference from the other chemical, and the difference in the absorbance of the relevant compound is proportional to its concentration. The determination of NMV depended on the difference in absorbance between the chosen wavelengths (231 and 248 nm), as the RIT absorbances at the same wavelengths were equal. Similarly, NMV had the same absorbance values at 251 and 272 nm; consequently, the difference between the absorbance values at these two wavelengths was selected for RIT estimation. The calibration curves for NMV and RIT were plotted by considering the differences in absorbance at the chosen wavelengths for each of the two compounds and their corresponding concentrations.

### Methods validation

Our suggested methods were validated in accordance with ICH standards [29] as follows:

#### Linearity

The derivative values calculated with ^1^D and ^2^D or the difference in absorbance determined with DWZ, each selected at the predetermined points, were related to the corresponding concentrations of NMV and RIT. For all the suggested techniques, the regression coefficient (r) values were greater than 0.999, indicating a high degree of linearity (Table 1). In addition, the standard deviations of the intercept (Sa) and the slope (Sb) were low (Table 1).

**Table 1:** Analytical performance data of the calibration graphs for the determination of NMV, and RIT by the proposed methods

Analyte	NMV			RIT		
Method	^1^D	^2^D	DWZ	^1^D	^2^D	DWZ
Wavelength (nm)	222	222	∆λ (231–248)	253	253	∆λ (251–272)
Linearity range (µg/mL)	10–250			10–250		
LOD (µg/mL)^a^	1.37	2.65	2.69	3.22	2.43	2.75
LOQ (µg/mL)^b^	4.14	8.04	8.14	9.75	7.35	8.35
Regression coefficient	0.9999	0.9998	0.9998	0.9996	0.9998	0.9997
Slope	0.0009	0.0004	0.0010	0.0010	0.0008	0.0028
S_b_ of slope^c^	1.03E − 05	9.74E−06	2.32E−05	2.74E−05	1.58E−05	6.39E−05
Intercept	− 4.44E − 04	3.47E−18	− 2.93E−03	2.70E−03	2.11E−03	4.56E−03
S_a_ of intercept^d^	3.73E-04	3.53E−04	8.41E−04	9.75E−04	5.75E−04	2.32E−03

^a^Limit of detection

^b^Limit of quantitation

^c^Standard deviation of the slope

^d^Standard deviation of the intercept

#### Limit of quantitation (LOQ) and limit of detection (LOD)

The following equations were used to determine the LODs and LOQs of NMV and RIT in accordance with ICH recommendations [26]:

The formulas for calculating the LOD and LOQ were $\frac{3.3 \sigma }{S}$ and $\frac{10 \sigma }{S}$, respectively, where S is the slope of the calibration graph and σ is the standard deviation of the y‐intercepts of the regression line (S_a_).

The low values of the LOD and LOQ (Table 1) verified the high sensitivity of the derived methods. The most sensitive technique was ^1^D for NMV and ^2^D for RIT, with LOD values of 1.37 and 2.43 μg/mL and LOQ values of 4.14 and 7.35 μg/mL for NMV and RIT, respectively.

#### Trueness

The trueness of the proposed techniques was evaluated by assaying mixtures of NMV and RIT prepared to cover the linearity range of each studied compound and varying the ratios, as discussed in Sect. "Preparation and analysis of laboratory-prepared mixtures". The excellent mean % recoveries of the suggested approaches shown in Table 2 suggest superb trueness.

**Table 2:** Assay results for the determination of NMV, and RIT in synthetic mixtures

% Recovery^a^ ± RSD						
Analyte	NMV			RIT		
Method	^1^D	^2^D	DWZ	^1^D	^2^D	DWZ
Mix 1^b^	99.84 ± 0.38	100.09 ± 0.42	100.22 ± 0.59	99.78 ± 1.39	100.00 ± 0.83	99.76 ± 1.19
Mix 2^c^	100.00 ± 1.18	98.67 ± 0.58	99.73 ± 0.23	99.20 ± 2.00	99.83 ± 0.58	100.19 ± 0.66
Mix 3^d^	99.51 ± 1.13	100.28 ± 1.27	99.44 ± 0.51	99.56 ± 1.11	99.44 ± 1.00	100.58 ± 0.46
Average	99.78 ± 0.25	99.68 ± 0.88	99.80 ± 0.40	99.51 ± 0.29	99.76 ± 0.29	100.18 ± 0.41

^a^The average of the three determinations

^b^Mix 1 containing 225 NMV and 75 RIT μg/mL

^c^Mix 2 containing 100 NMV and 100 RIT μg/mL

^d^Mix 3 containing 15 NMV and 45 RIT μg/mL

#### Precision

Solutions of three different volumes of NMV and RIT, mentioned in Sect. "Preparation and analysis of laboratory-prepared mixtures", were analysed in triplicate on the same day and on three successive days to investigate the intraday and interday precision, respectively. The concentrations obtained from the relevant regression equations were utilized to calculate a % RSD not exceeding 2%, demonstrating the excellent precision of the provided approaches (Table 2).

#### Selectivity

Within the linearity limits, selectivity was assessed via various laboratory-prepared combinations containing different ratios of NMV and RIT (Table 2). The results demonstrated very good % mean recoveries (100% ± 2) for NMV and RIT, with relative SD values of ± 2% in ^1^D, ^2^D, and DWZ for both NMV and RIT.

### Statistical comparison between the developed methods

A one-way ANOVA [30] test was performed on three synthetic mixtures of both drugs, with n = 3, to confirm the outcomes of the proposed procedures. Snedecor’s F values were therefore calculated and compared to the commonly used value (P = 0.05). Table 3 shows that while the experimental or computed F values (0.04 and 3.01 for NMV and RIT, respectively) did not exceed the tabulated values (5.14) in the analysis of variance, there was no appreciable difference between the approaches.

**Table 3:** Summary of assay results and one–way analysis of variance (ANOVA) for the determination of NMV and RIT by ^1^D, ^2^D and DWZ

	Source of Variation	SS	Df	MS	F	P-value	F crit
NMV	Between groups	0.02	2	0.01	**0.04**	0.96	**5.14**
	Within groups	1.98	6	0.33			
	Total	2.01	8				
RIT	Between groups	0.68	2	0.34	**3.01**	0.12	**5.14**
	Within groups	0.67	6	0.11			
	Total	1.35	8				

Bold illustrates important values

### Application of the validated methods in the assay of tablet dosage forms

The established methodologies were successfully used to quantify NMV and RIT in PAXLOVID^®^ tablet doses. Acceptable values for the standard deviation and percentage recoveries (%) demonstrated the precision and accuracy of the assay results (Table 4). The analytical performance of the validated procedures, as previously mentioned, confirms their applicability for the routine analysis of NMV and RIT in their copacked tablets, which requires little sample preparation and yields acceptable analytical results.

**Table 4:** Assay results for the determination of NMV, and RIT in dosage form

% Recovery^a^						
Analyte	NMV			RIT		
Method	^1^D	^2^D	DWZ	^1^D	^2^D	DWZ
Mean ±	99.86 ±	100.56 ±	100.02 ±	99.54 ±	100.08 ±	99.41 ±
SD	0.50	0.26	0.16	0.85	1.09	1.10

^a^The average of the three determinations

## Analytical and ecological review of methods for analysing NMV and RIT

### Analytical review

Few methods have been reported for analysing NMV and RIT simultaneously. These methods are versatile and cover different chromatographic techniques, as summarized in Table 5. Chromatographic methods include chromatographic and electrophoretic techniques. The first published method was UPLC–MS/MS [13], which has optimum sensitivity (nanorange) and the lowest injection volume (5 µL). In addition, this method, owing to its triple quadrupole MS, guarantees high specificity in selective reaction monitoring mode, making the analysis in plasma reliable. On the other hand, two HPLC‒DAD methods for analysing NMV and RIT in tablets have been reported [15, 16]. The first method [15] was more sensitive than the second method [16]. However, the second study analysed both drugs in the presence of their degradation products and with a shorter run time than did the first study (7 min instead of 10 min). In addition to liquid chromatography, one thin-layer chromatography method was reported to analyse the studied drugs in tablets and human plasma [14]. In this method, a small volume of β-CD was added to the mobile phase to increase the resolution between NMV and RIT. Unlike most TLC methods, the reported TLC method is highly sensitive compared with the reported HPLC methods for the same drugs [15, 16]. On the other hand, the only reported CE method for analysing NMV and RIT is micellar electrokinetic chromatography using 25 mM SDS [16]. This method analysed both drugs in the presence of their degradation products with high sensitivity compared with the HPLC method reported in the same paper [16]. Notably, acetonitrile has been used for the efficient extraction of NMV and RIT from biological media [13, 14]. To our knowledge, our method is the first spectrophotometric method that successfully analyses NMV and RIT with moderate sensitivity via different simple mathematical techniques. Moreover, our spectrophotometric method offers several advantages over chromatographic techniques, including reduced solvent usage, lower energy consumption, simplified sample preparation, cost-effectiveness, high-throughput potential, alignment with green chemistry principles, and routine analysis and screening. It requires lower solvent volumes and minimal extraction or dilution, reducing solvent waste and environmental impact. In general, spectrophotometric instruments also consume less energy, making them simpler and quicker to operate. They also offer high-throughput potential, making them ideal for pharmaceutical laboratories. Overall, spectrophotometric methods offer practical solutions without the complexity of chromatographic techniques. Thus, the recent method is considered the most ideal among all reported methods for quality control purposes.

**Table 5:** Different analytical methods for the determination of NMV and RIT in different matrices

Method	Sample Type	Injection volume	Stationary phase	Mobile phase/ Solvent	Linearity range μg/mL	LOQ μg/mL		Detector	Refs.
Spectrophotometry	Tablets	–	–	EtOH	10–250	**NMV**	^1^D 4.14 ^2^D 8.04 DWZ8.14	UV	This work
						**RIT**	^1^D 9.75 ^2^D 7.35 DWZ8.35		
TLC-DAD	Tablets and spiked human plasma	10 µL	TLC aluminum silica gel plates	A: MeOH B: H_2_O C: 2% urea solution of β-cyclodextrin (40:10:0.5)	1–5	**NMV** 0.21 **RIT** 0.13		215 nm	[14]
HPLC–DAD	Tablets	20 µL	C18 column (4.6 × 250 mm, 5 μm particle size)	A: 20% Water B: 80% EtOH Flow rate: 1 mL/min Run time 10 min	1–20	**NMV** 0.6 **RIT** 0.96		215 nm	[15]
CE-DAD	Tablets and in presence of degradation products	Inject at 50 mbar pressure, for 17 s	silica capillary (50 cm effective length × 50 μm id)	50 mM borate buffer pH 9.2 with 25 mM sodium lauryl sulfate Run time 7 min	**NMV** 10–200 **RIT** 5–100	**NMV** 2.78 **RIT** 1.95		210 nm	[16]
HPLC–DAD	Tablets and in presence of degradation products	20 µL	C18 (4.6 × 250 mm, 5 μm particle size)	A: 50% 50 mM ammonium acetate buffer at pH 5 B: 50% ACN Flow rate: 1 mL/min Run time 7 min	**NMV** 10–200 **RIT** 5–100	**NMV** 2.22 **RIT** 1.23		210 nm	[16]
UPLC-MS/MS	Human plasma	5 µL	BDS Hypersil C18 column (4.6 × 100 mm, 2.4 μm)	Gradient elution A: 0.1% formic acid H_2_O B: 0.1% formic acid MeOH Flow rate: 0.8 mL/min Run time 7 min	**NMV** 50–5000 **RIT** 10–1000 ng/mL	**NMV** 50 **RIT** 10 ng/mL		**NMV** m/z 500.2→ 319.0, **RIT** m/z 721.3 →267.8	[13]

Bold illustrates important values

### Ecological review

The environmental friendliness and sustainability of an analytical approach are generally evaluated via meticulous greenness and whiteness tools. The choice of solvent is especially important in determining the greenness of a method, so two green assessment approaches were first implemented to evaluate the solvent used: the green solvent selection tool (GSST) and the spider diagram. In addition, the whole methodology was assessed via several approaches based on the penalization strategy or the colour-coded strategy. The greenness of the proposed methods was assessed via the analytical eco-scale, GAPI, Hexagon, and AGREE. Additionally, two assessment tools, the RGB and hexagon methods, were used to assess the sustainability of the method.

#### Assessment of the greenness of solvents

##### Green solvent selection tool (GSST)

Qualified companies would likely agree that a certified green solvent should have a low health risk, high safety, and negligible environmental impact throughout its whole life cycle.

Many pharmaceutical corporations, including Pfizer, Sanofi, and GlaxoSmithKline (GSK), have criteria for selecting appropriate green sustainable solvents to achieve sustainability in the pharmaceutical sector. The GSK Solvent Sustainability Guidelines offer solvent sustainability recommendations to help determine which solvents to use. The advantages and disadvantages of various solvents are based on the information provided in solvent safety data sheets (SDSs).

Christian Larsen’s research created a new chemometric tool to choose a green and sustainable solvent (G) by evaluating the greenness score provided by the GSK Solvent Sustainability Guidelines, which provides a quantitative measurement of solvents on the basis of a wide range of different criteria in the form of a composite score [26].

G = ∜(H × S × E × W), where (G) represents the numerical value of the fourth root created by multiplying four major sustainable factors: health (H), safety (S), environment (E), and waste disposal (W). More information is accessible in this manuscript [26], and a free solvent calculator is available at this link: http://green-solvent-tool.herokuapp.com/

A lower G indicates that the solvent's qualities are not sustainable, whereas a higher score indicates that the solvent's features are desirable in terms of sustainability and greenness. All values are between 1 and 10.

This score reflects the analyser’s endeavor to produce a more environmentally friendly substitute for solvents with high G values that can be employed in the analytical procedure. The GSST provides a simple, quick, and free tool for analysing solvents in compliance with the GSK Solvent Sustainability Guidelines, and it was utilized for the first time in researching the sustainability of solvents used in analytical techniques.

When different solvents are compared, the green solvent selection tool is simple to use and swift to obtain results online. However, not all chemical solvents are available with this tool, as it is limited to a small number of commonly used solvents and relies on evaluating sustainability according to a specific mathematical equation.

When comparing the acquired G values of the solvents, which were calculated directly online via the GSST, with the different methods reported for the analysis of NMV and RIT, we compared the solvents used in these methods. The proposed UV methods employed EtOH as the solvent. UPLC–MS/MS [13] uses MeOH and acidified water, whereas HPLC–DAD methods use either EtOH and water [15] or ACN and buffer [16] as mobile phases. Additionally, the HPTLC method was developed using MeOH and water [14]. The organic modifier, whenever it exists in chromatographic methods, is to blame for the G value, whereas water does not affect the evaluation of greenness. Nonetheless, the organic modifier is the key factor in determining greenness solvent values in analytical approaches. Unlike other chromatographic techniques, the CE method can utilize water solely to prepare the buffer without any organic modifier, as reported in our previous paper [16].

**Fig. 6:**
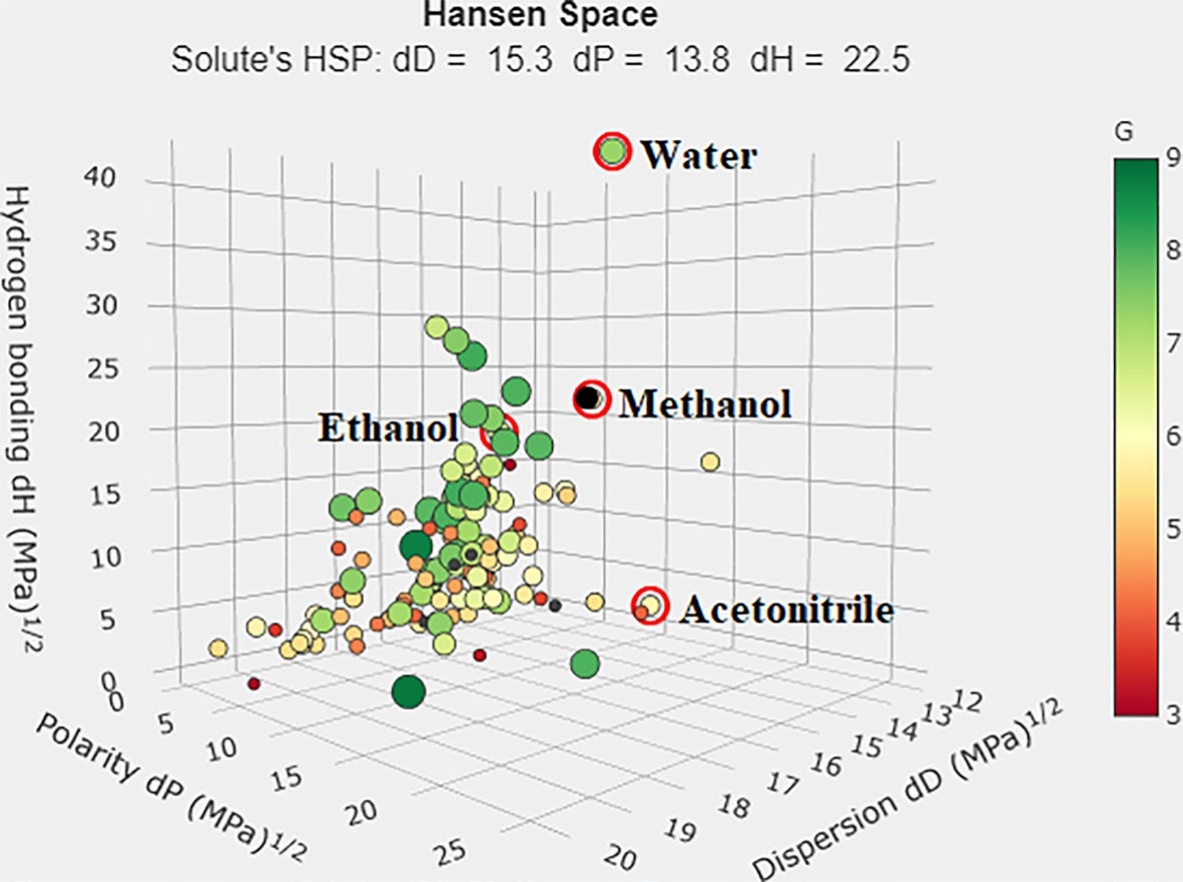
*Hansan* space for solvent selection showing the G values of water, EtOH, MeOH and ACN

The G values shown in Fig. 6 indicate that water is superior (G score of 7.3 with the following category scores: W = 3.7, H = 9.5, E = 8.9, and S = 8.9). In the second place comes EtOH (G of ethanol = 6.6, with the following category scores: W = 4.2, H = 8.9, E = 6.7, and S = 7). Surprisingly, ACN and MeOH obtained the same G score (G = 5.8) but different category scores: ACN: W = 2.8, H = 5.9, E = 8.9, and S = 7; MeOH: W = 4.0, H = 4.9, E = 8.4, and S = 7.1. Therefore, using the GSST, our previously reported CE method was superior [16], with its performance followed by those of the spectrophotometric methods proposed in this study and the reported HPLC methods [15], and finally, the methods utilizing either MeOH or ACN exhibited the worst performances [13, 14, 16].

##### Spider diagram for the assessment of the greenness index

The reagents used in the proposed spectrophotometric methods and the reported chromatographic methods were thoroughly evaluated with the aid of the greenness index with a spider diagram. While our spectrophotometric methods use EtOH, the reported LC methods use EtOH [15], ACN [16] or MeOH [13] along with water or acidified water. On the other hand, the reported HPTLC method [14] utilized MeOH, and the CE method used buffer dissolved in water [16].

This tool is based on data from SDSs, which provide details about the properties of a solvent and how they affect the SHE throughout the entire process. A visual representation of the overall degree of sustainability of the chemicals used was produced by integrating five subcategories of evaluation criteria (health impact, overall characteristics, odour, fire safety, and stability) in a hierarchical spider diagram. On the basis of these criteria, scores between − 5 and + 5 were obtained. Additional spider charts that provide additional details on each of the five previously mentioned subcategories are available. For the five aforementioned subgroups, many chemical reagents do not provide all of the information required on an SDS; hence, these missing data were given a score of zero throughout the computation. All of the missing data and the accessible data needed to calculate the greenness index values are made available in a data table called the greenness index table. The degree of integrity in the greenness assessment is shown in these references. [22–25]

**Fig. 7:**
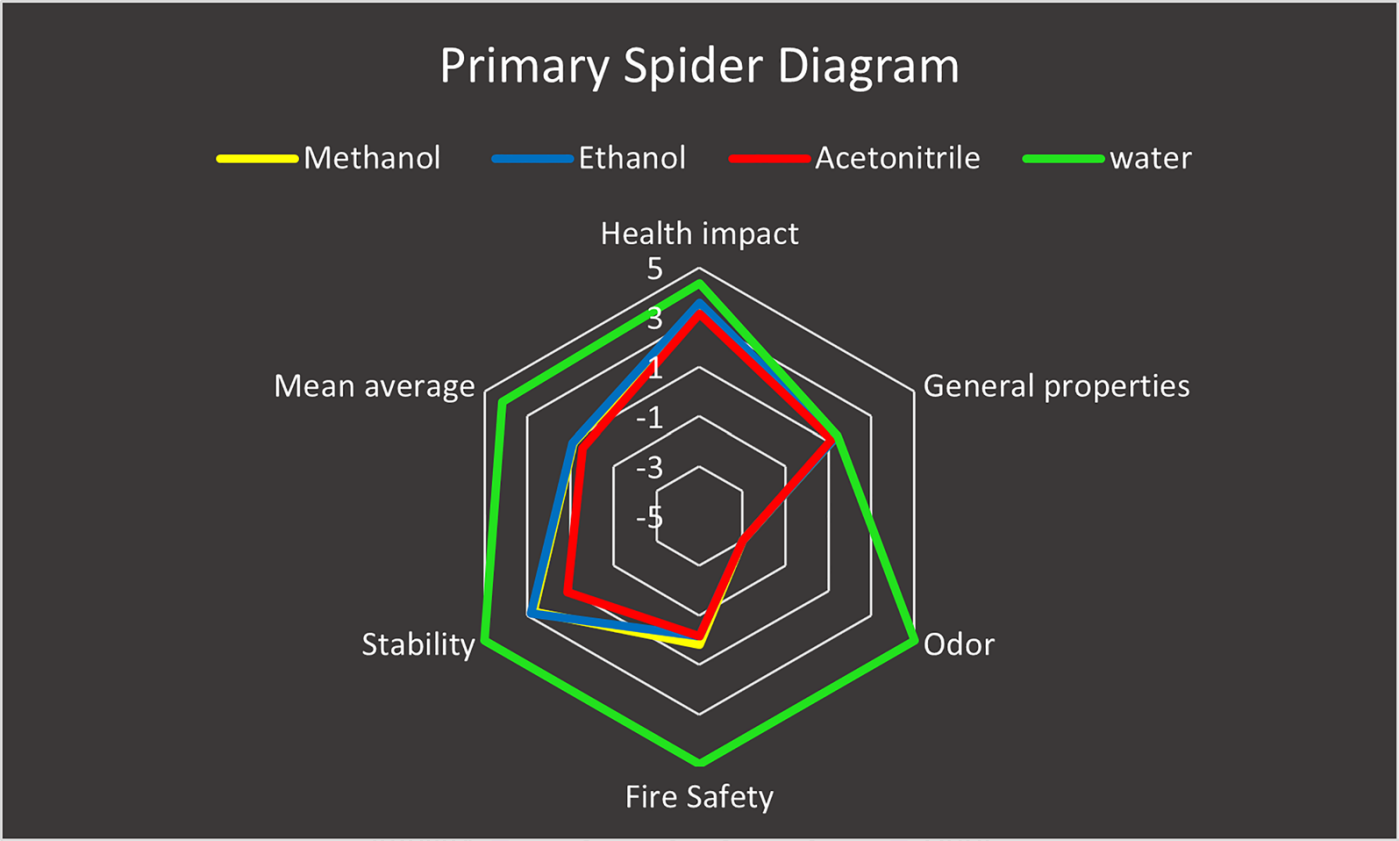
Primary spider diagram for water, EtOH, MeOH and ACN

**Fig. 8:**
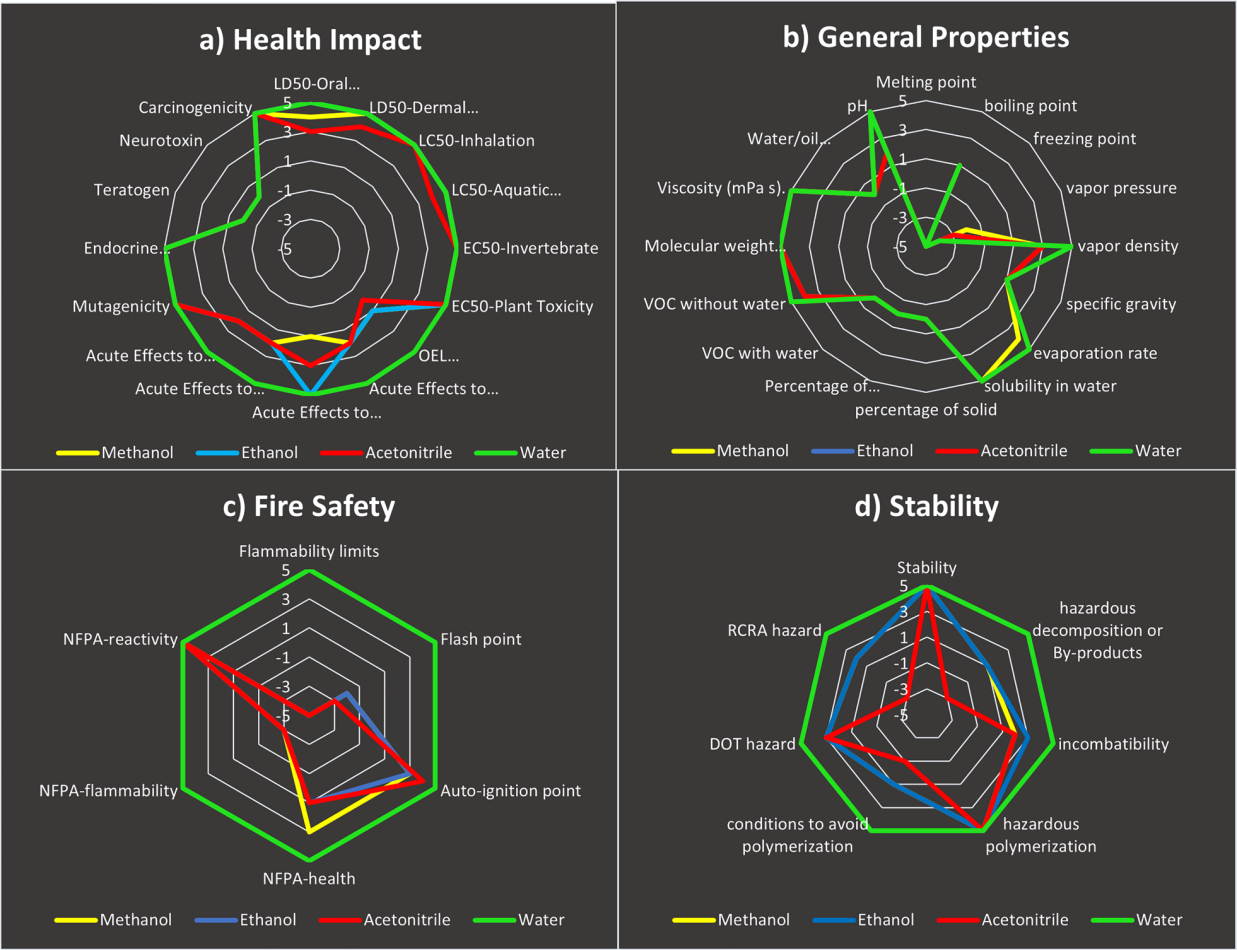
Secondary spider diagram for **A**- health impact, **B**- stability, **C**- general properties, and **D**- fire safety for water, EtOH, MeOH and ACN

To provide a comprehensive analysis of the solvents used, the greenness index was determined via a spider diagram for both the proposed spectrophotometric method and the reported methods. The primary spider chart in Fig. 7 shows that water's overall greenness is at the top, followed by that of EtOH and MeOH, with EtOH demonstrating slightly higher overall greenness, whereas ACN has the lowest point sums, indicating a decrease in the safe region. The supplementary spider charts in Fig. 8a–d show the supporting data for the other scores. The average scores and percentages of significant data for the solvents currently in use are shown in Table 6 for the greenness index. This spider method enables visual reagent evaluation, making it easy to compare. Since the proposed spectrophotometric method only uses EtOH and the reported CE method uses buffer [16], the reported chromatographic methods utilize either ACN, MeOH or EtOH along with buffers or water; however, buffer and water are regarded safe rather than harmful solvents. As a result, if the organic modifier is present, it is to blame for the hazard score, whereas the buffer or water can be deemed to have no effect on the evaluation of greenness, as mentioned in the GSST.

**Table 6:** Greenness Index Table for solvents: Water, EtOH, MeOH and ACN

	Water score	Available information	EtOH score	Available information	MeOH score	Available information	CAN score	Available information
Health impact	4.38	100	3.56	100	3.25	100	3.13	100
General properties	1.44	87.5	1.31	87.5	1.31	87.5	1.13	87.5
Odor	5	100	− 3	100	− 3	100	− 3	100
Fire safety	5.00	100	− 0.17	100	0.17	100	− 0.17	100
Stability	5.00	100	2.86	100	2.71	100	1.14	100
Mean average	4.16	97.50	0.91	97.50	0.89	97.50	0.45	97.50

The previous spider diagram results and the greenness index table show that the EtOH solvent, with an average score of 0.91, is safer for the environment and human health, and it is preferable to use UV methods in light of green chemistry. The greenness index method is more difficult to apply than is the GSST approach. Adopting the spider greenness index method is more challenging since it necessitates independent work and analysis of several SDSs to obtain the most relevant data. Moreover, if missing data are set to zero for one of the solvents, it is not appropriate to provide the same information for another solvent with a value other than zero. If so, this value will give the second solvent either positive or negative weight depending on the accessibility of the data. In our work, the missing data for one solvent were set to zero for all the other solvents to avoid bias.

Nonetheless, this method uses secondary spider charts to describe the associated subpoints for every greenness criterion, resulting in a visual representation of the solvent greenness comparability. The research offers greenness points for each solvent used, and the greenness table index offers a high-level summary.

#### Assessment of the greenness and sustainability of the proposed analytical method

##### Penalization strategy

A preliminary evaluation of the devised method was performed using the earliest and most familiar analytical indicator for going green, the analytical eco-scale (ESA) tool [31]. This assessment relies mostly on a penalization technique that assigns penalty points (which are deducted from 100, the ideal score). The score indicates how closely the performance adheres to ideality. The ESA score (93) revealed a high level of obedience to GAC principles (Table 7).

**Table 7:** Greenness assurance of the suggested spectrophotometric methods via versatile metrics

Reagents/instruments	Penalty points (PPs)	GAPI	AGREE	Hexagon
Ethanol	4			
Energy of spectrophotometer	0			
Occupational hazard	0			
waste	3			
PPs	**7**			
Eco-scale score	**93**			

Bold illustrates important values

The penalization notion is also implemented via the hexagon tool [32]. A more complicated tool, however, provides multicriteria assessment, including evaluation of analytical performance, implications for the environment and economy, and sustainability. With a pictogram indicating low penalty points (2 ones and 5 zeros), the spectrophotometric method demonstrated green and sustainable performance (Table 7).

##### Colour-coded strategy

The most user-friendly and applicable tools with the final results determined by colour are the newly booming RGB12 and AGREE, and the oldest among them is GAPI. The developed method was examined and integrated into our previously reported comparative study of greenness and whiteness via RGB12 and AGREE tools [16, 33].

The GAPI model is a semiquantitative tool with characteristic pentagrams covering 15 parameters for evaluating the whole analytical methodology. The evaluation includes sample preparation, the solvents and instruments used, and the central circle for qualification and quantification of the method. The original article presents an explanation of the GAPI parameters together with their corresponding colour codes (green, yellow and red) [34] and clarifies that as the number of green sectors increases, the environmental impact decreases, indicating a greener method. The implemented spectrophotometric method results in many green areas with only one red area, as illustrated in Table 7.

**Fig. 9:**
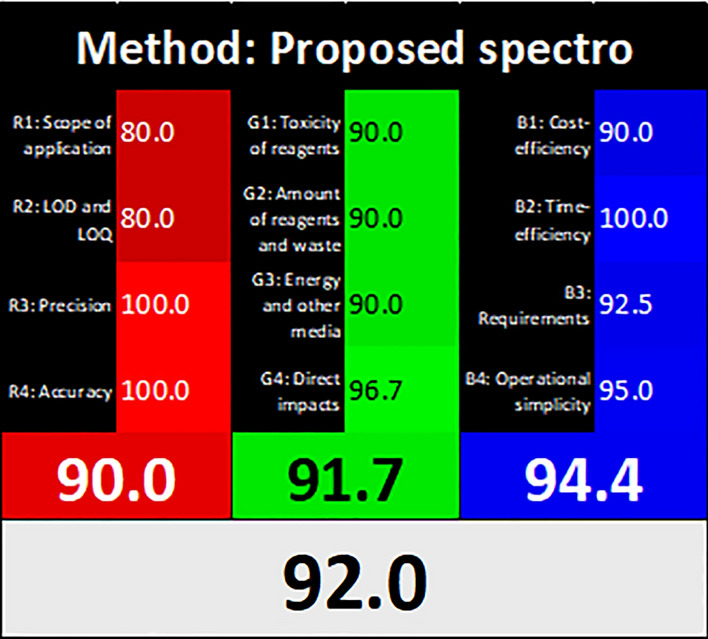
RGB12 profile and score of the proposed method

Recently, the integration of validation criteria, environmental benignity, and functionality has been fully covered by the white analytical chemistry paradigm. In this context, RGB12 is the latest trend in multicriteria assessment tools. A ready-to-use Excel worksheet is composed of three essentially coloured compartments (red, green and blue) that are linked together through addition and arithmetic means functions. The net result is the degree of whiteness percentage of the whole methodology [28]. The %whiteness of the method is 92%, as shown in Fig. 9. These results were juxtaposed to the comparative table in our recently published whiteness comparative study [16] and were rechecked to rank the suggested method. This score place the method in the fourth place, with a slight difference from the reported HPLC methods (second [16] and third places [15]). Although the spectrophotometric method is greener than the HPLC method because of the use of a green solvent (EtOH), it lacks automation, and the sample size is large (2 ml).

Since 2020, AGREE has been the most commonly used metric in the assessment of greenness in pharmaceuticals. A downloadable calculator (https://mostwiedzy.pl/AGREE) that fulfils the twelve GAC principles was applied. These parameters are filled and calculated, and the net result is given automatically with the corresponding grade of colour [35]. According to our aforementioned comparative study, comparing with the previously published chromatographic techniques shows that the spectrophotometric method presented in the study ranks second, following the MEKC method, as shown in Table 7.

## Conclusion

In this study, simple, sustainable, and earth-friendly spectrophotometric methods were investigated and fully validated for the simultaneous quantification of RIT and NMV in their bulk powder, synthetic admixture, and copacked tablet forms. Notably, this study is the first spectrophotometric analysis of the mixture under study. Notably, the spectrophotometric technique eschews the demerits of conventional chromatographic techniques. It offers ease of use, time, and cost efficiency. The greenness assessment protocol using several strategies, namely, solvent evaluation, penalization strategies and colour-coded strategies, illustrated the high greenness attributes of the proposed methods. Highlighted by the preceding considerations, the explored methods are highly recommended for quality control purposes.

## Data Availability

The corresponding author can provide the datasets used and/or analysed for this work upon reasonable request.

## Abbreviations

NMV: Nirmatrelvir
RIT: Ritonavir
LOD: Limit of detection
LOQ: Limit of quantification
GAPI: Green analytical procedure index
AGREE: Analytical greenness metric
DWZ: Dual-wavelength zero-order method
NEMI: National environmental method Index

## References

[1] Poduri R, Joshi G, Jagadeesh G. Drugs targeting various stages of the SARS-CoV-2 life cycle: exploring promising drugs for the treatment of Covid-19. Cell Signal. 2020;74: 109721. 32711111 10.1016/j.cellsig.2020.109721 PMC7375293

[2] Huang C, Wang Y, Li X, Ren L, Zhao J, Hu Y, Zhang L, Fan G, Xu J, Gu X, Cheng Z, Yu T, Xia J, Wei Y, Wu W, Xie X, Yin W, Li H, Liu M, Xiao Y, Gao H, Guo L, Xie J, Wang G, Jiang R, Gao Z, Jin Q, Wang J, Cao B. Clinical features of patients infected with 2019 novel coronavirus in Wuhan, China. Lancet. 2020;395(10223):497–506. 31986264 10.1016/S0140-6736(20)30183-5 PMC7159299

[3] Pan L, Mu M, Yang P, Sun Y, Wang R, Yan J, Li P, Hu B, Wang J, Hu C, Jin Y, Niu X, Ping R, Du Y, Li T, Xu G, Hu Q, Tu L. Clinical characteristics of COVID-19 patients with digestive symptoms in hubei, china: a descriptive, cross-sectional, multicenter study. Am J Gastroenterol. 2020;115(5):766–73. 32287140 10.14309/ajg.0000000000000620 PMC7172492

[4] Edwards K, Hussain I. Two cases of severe autoimmune thyrotoxicosis following SARS-CoV-2 infection. J Investig Med High Impact Case Rep. 2021;9:23247096211056496. 34844465 10.1177/23247096211056497 PMC8640318

[5] Baig AM, Sanders EC. Potential neuroinvasive pathways of SARS-CoV-2: Deciphering the spectrum of neurological deficit seen in coronavirus disease-2019 (COVID-19). J Med Virol. 2020;92(10):1845–57. 32492193 10.1002/jmv.26105 PMC7300748

[6] Du Y, Tu L, Zhu P, Mu M, Wang R, Yang P, Wang X, Hu C, Ping R, Hu P, Li T, Cao F, Chang C, Hu Q, Jin Y, Xu G. Clinical features of 85 fatal cases of COVID-19 from Wuhan a retrospective observational study. Am J Respir Crit Care Med. 2020;201(11):1372–9. 32242738 10.1164/rccm.202003-0543OC PMC7258652

[7] Torres I, Artaza O, Profeta B, Alonso C, Kang J. COVID-19 vaccination: returning to WHO’s Health For All. Lancet Glob Health. 2020;8(11):e1355–6. 32986981 10.1016/S2214-109X(20)30415-0 PMC7518851

[8] Owen DR, Allerton CMN, Anderson AS, Aschenbrenner L, Avery M, Berritt S, Boras B, Cardin RD, Carlo A, Coffman KJ, Dantonio A, Di L, Eng H, Ferre R, Gajiwala KS, Gibson SA, Greasley SE, Hurst BL, Kadar EP, Kalgutkar AS, Lee JC, Lee J, Liu W, Mason SW, Noell S, Novak JJ, Obach RS, Ogilvie K, Patel NC, Pettersson M, Rai DK, Reese MR, Sammons MF, Sathish JG, Singh RSP, Steppan CM, Stewart AE, Tuttle JB, Updyke L, Verhoest PR, Wei L, Yang Q, Zhu Y. An oral SARS-CoV-2 M(pro) inhibitor clinical candidate for the treatment of COVID-19. Science. 2021;374(6575):1586–93. 34726479 10.1126/science.abl4784

[9] Hammond J, Leister-Tebbe H, Gardner A, Abreu P, Bao W, Wisemandle W, Baniecki M, Hendrick VM, Damle B, Simón-Campos A, Pypstra R, Rusnak JM. Oral nirmatrelvir for high-risk, nonhospitalized adults with COVID-19. N Engl J Med. 2022;386(15):1397–408. 35172054 10.1056/NEJMoa2118542 PMC8908851

[10] Abdelnabi R, Foo CS, Jochmans D, Vangeel L, De Jonghe S, Augustijns P, Mols R, Weynand B, Wattanakul T, Hoglund RM, Tarning J, Mowbray CE, Sjö P, Escudié F, Scandale I, Chatelain E, Neyts J. The oral protease inhibitor (PF-07321332) protects Syrian hamsters against infection with SARS-CoV-2 variants of concern. Nat Commun. 2022;13(1):719. 35169114 10.1038/s41467-022-28354-0 PMC8847371

[11] Wan KX, Potts D, Gonzalez P, Smith I, Shi H, Kavetska O. Bioanalytical method validation and sample analysis for nirmatrelvir in dried blood collected using the Tasso-M20 device. Bioanalysis. 2022;14(20):1305–15. 36541270 10.4155/bio-2022-0167

[12] Aboras SI, Maher HM. Green adherent degradation kinetics study of Nirmatrelvir, an oral anti-COVID-19: characterization of degradation products using LC-MS with insilico toxicity profile. BMC Chem. 2023;17(1):23. 36932440 10.1186/s13065-023-00928-z PMC10020773

[13] Liu C, Zhu M, Cao L, Boucetta H, Song M, Hang T, Lu Y. Simultaneous determination of nirmatrelvir and ritonavir in human plasma using LC-MS/MS and its pharmacokinetic application in healthy Chinese volunteers. Biomed Chromatogr BMC. 2022;36(11): e5456. 35881032 10.1002/bmc.5456

[14] Imam MS, Abdelazim AH, Batubara AS, Gamal M, Almrasy AA, Ramzy S, Khojah H, Hasanin THA. Simultaneous green TLC determination of nirmatrelvir and ritonavir in the pharmaceutical dosage form and spiked human plasma. Sci Rep. 2023;13(1):6165. 37061601 10.1038/s41598-023-32904-x PMC10105527

[15] Imam MS, Batubara AS, Gamal M, Abdelazim AH, Almrasy AA, Ramzy S. Adjusted green HPLC determination of nirmatrelvir and ritonavir in the new FDA approved co-packaged pharmaceutical dosage using supported computational calculations. Sci Rep. 2023;13(1):137. 36599900 10.1038/s41598-022-26944-y PMC9811874

[16] Elbordiny HS, Alzoman NZ, Maher HM, Aboras SI. Tailoring two white chromatographic platforms for simultaneous estimation of ritonavir-boosted nirmatrelvir in their novel pills: degradation, validation, and environmental impact studies. RSC Adv. 2023;13(38):26719–31. 37681051 10.1039/d3ra04186g PMC10481124

[17] Abdelhameed RM, Hammad SF, Abdallah IA, Bedair A, Locatelli M, Mansour FR. A hybrid microcrystalline cellulose/metal-organic framework for dispersive solid phase microextraction of selected pharmaceuticals: a proof-of-concept. J Pharm Biomed Anal. 2023;235: 115609. 37557067 10.1016/j.jpba.2023.115609

[18] Mansour FR, Abdelhameed RM, Hammad SF, Abdallah IA, Bedair A, Locatelli M. A microcrystalline cellulose/metal-organic framework hybrid for enhanced ritonavir dispersive solid phase microextraction from human plasma. Carbohydr Polymer Technol Appl. 2024;7: 100453.

[19] Karpińska J. Basic principles and analytical application of derivative spectrophotometry. Houston: InTech; 2012.

[20] Fawzy MG, Mostafa AA, Shalaby A, Sayed RA. Green-assisted spectrophotometric techniques utilizing mathematical and ratio spectra manipulations to resolve severely overlapped spectra of a cardiovascular pharmaceutical mixture. Spectrochim Acta Part A Mol Biomol Spectrosc. 2023;295: 122588. 10.1016/j.saa.2023.122588 36934596

[21] Elbordiny HS, Elonsy SM, Daabees HG, Belal TS. Sustainable quantitative determination of allopurinol in fixed dose combinations with benzbromarone and thioctic acid by capillary zone electrophoresis and spectrophotometry: validation, greenness and whiteness studies. Sustain Chem Pharm. 2022;27: 100684.

[22] Abou-Taleb NH, El-Enany NM, El-Sherbiny DT, El-Subbagh HI. Spider diagram and Analytical GREEnness metric approach for assessing the greenness of quantitative 1H-NMR determination of lamotrigine: Taguchi method based optimization. Chemom Intell Lab Syst. 2021;209: 104198.

[23] Shen Y, Lo C, Nagaraj DR, Farinato R, Essenfeld A, Somasundaran P. Development of Greenness Index as an evaluation tool to assess reagents: evaluation based on SDS (safety data sheet) information. Miner Eng. 2016;94:1–9.

[24] Lotfy HM, Obaydo RH, Nessim CK. Spider chart and whiteness assessment of synergistic spectrophotometric strategy for quantification of triple combination recommended in seasonal influenza—detection of spurious drug. Sustain Chem Pharm. 2023;32: 100980.

[25] Kayali Z, Obaydo RH, Alhaj Sakur A. Spider diagram and sustainability evaluation of UV-methods strategy for quantification of aspirin and sildenafil citrate in the presence of salicylic acid in their bulk and formulation. Heliyon. 2023;9(4): e15260. 37123917 10.1016/j.heliyon.2023.e15260 PMC10130774

[26] Larsen C, Lundberg P, Tang S, Ràfols-Ribé J, Sandström A, Mattias Lindh E, Wang J, Edman L. A tool for identifying green solvents for printed electronics. Nat Commun. 2021;12(1):4510. 34301943 10.1038/s41467-021-24761-x PMC8302666

[27] Welch CJ, Wu N, Biba M, Hartman R, Brkovic T, Gong X, Helmy R, Schafer W, Cuff J, Pirzada Z, Zhou L. Greening analytical chromatography. TrAC Trends Anal Chem. 2010;29(7):667–80.

[28] Nowak PM, Wietecha-Posłuszny R, Pawliszyn J. White analytical chemistry: an approach to reconcile the principles of green analytical chemistry and functionality. TrAC Trends Anal Chem. 2021;138:116223.

[29] International Conference on Harmonisation (ICH). ICH, validation of analytical procedures: text and methodology. Geneva: ICH; 2005. p. 2005.

[30] Miller, J. N.; Miller, J. C. In Statistics and chemometrics for analytical chemistry, 6th Edition, 2010.

[31] Gałuszka A, Migaszewski ZM, Konieczka P, Namieśnik J. Analytical eco-scale for assessing the greenness of analytical procedures. TrAC, Trends Anal Chem. 2012;37:61–72.

[32] Ballester-Caudet A, Campíns-Falcó P, Pérez B, Sancho R, Lorente M, Sastre G, González C. A new tool for evaluating and/or selecting analytical methods: Summarizing the information in a hexagon. TrAC, Trends Anal Chem. 2019;118:538–47.

[33] Locatelli M, Kabir A, Perrucci M, Ulusoy S, Ulusoy HI, Ali I. Green profile tools: current status and future perspectives. Adv Sample Preparation. 2023;6: 100068.

[34] Płotka-Wasylka J. A new tool for the evaluation of the analytical procedure: green analytical procedure index. Talanta. 2018;181:204–9. 29426502 10.1016/j.talanta.2018.01.013

[35] Pena-Pereira F, Wojnowski W, Tobiszewski M. AGREE—analytical GREEnness metric approach and software. Anal Chem. 2020;92(14):10076–82. 32538619 10.1021/acs.analchem.0c01887 PMC7588019

